# JAK/STAT and TGF-ß activation as potential adverse outcome pathway of TiO_2_NPs phototoxicity in *Caenorhabditis elegans*

**DOI:** 10.1038/s41598-017-17495-8

**Published:** 2017-12-19

**Authors:** Hunbeen Kim, Jaeseong Jeong, Nivedita Chatterjee, Carlos P. Roca, Dahye Yoon, Suhkmann Kim, Younghun Kim, Jinhee Choi

**Affiliations:** 1 School of Environmental Engineering, University of Seoul, 163 Seoulsiripdaero, Dongdaemun-gu, Seoul 02504 Korea; 2 Department of Bioscience, Aarhus University, 8600 Silkeborg, Denmark; 3 Autoimmune Genetics Laboratory, Department of Microbiology and Immunology, KU Leuven – University of Leuven, B-3000, Leuven, Belgium; 4 Department of Chemistry, Center for Proteome Biophysics and Chemistry Institute for Functional Materials, Pusan National University, Busan, 46241 Korea; 5 Department of Chemical Engineering, Kwangwoon University, 20 Kwangwoon-ro, Nowon-gu, Seoul 01897 Korea; 6 VIB Center for Brain and Disease Research, B-3000, Leuven, Belgium

## Abstract

Titanium dioxide nanoparticles (TiO_2_NPs) are widely used nanoparticles, whose catalytic activity is mainly due to photoactivation. In this study, the toxicity of TiO_2_NPs was investigated on the nematode *Caenorhabditis elegans*, with and without UV activation. Comparative analyses across the four treatments revealed that UV-activated TiO_2_NPs led to significant reproductive toxicity through oxidative stress. To understand the underlying molecular mechanism, transcriptomics and metabolomics analyses were conducted, followed by whole-genome network-based pathway analyses. Differential expression analysis from microarray data revealed only 4 DEGs by exposure to TiO_2_NPs alone, compared to 3,625 and 3,286 DEGs by UV alone and UV-activated TiO_2_NPs, respectively. Pathway analyses suggested the possible involvement of the JAK/STAT and TGF-ß pathways in the phototoxicity of TiO_2_NPs, which correlated with the observation of increased gene expression of those pathways. Comparative analysis of *C. elegans* response across UV activation and TiO_2_NPs exposure was performed using loss-of-function mutants of genes in these pathways. Results indicated that the JAK/STAT pathway was specific to TiO_2_NPs, whereas the TGF-ß pathway was specific to UV. Interestingly, crosstalk between these pathways was confirmed by further mutant analysis. We consider that these findings will contribute to understand the molecular mechanisms of toxicity of TiO_2_NPs in the natural environment.

## Introduction

In recent years, nanotoxicology research has been carried out using a wide range of model systems, mostly under well-controlled laboratory conditions. In the environment, however, most organisms face nanoparticle (NP) exposure simultaneously with various physical, chemical and biological stresses. Therefore, to better understand the adverse effect of NPs in the environment, exposure scenarios for nanotoxicity testing should reflect real environmental conditions. Various physical, chemical, and biological factors (i.e. temperature, UV, salinity, oxygen, chemicals, pathogens, etc.) can affect the response of organisms to NPs. TiO_2_NPs are widely used NPs, whose applications range from cosmetics to catalysts^1,2^. For their safe use, and because their catalytic activity is mainly due to photoactivation^3^, the toxicity of TiO_2_NPs should be studied under photoactivated conditions. However, although the phototoxicity of TiO_2_NPs has been studied before^4–6^, few studies have addressed the molecular mechanisms of phototoxicity in a comprehensive way.

Systems toxicology approaches using profiling techniques based on multi-OMICS assays (i.e. transcriptomics, proteomics, and metabolomics) have proven to be effective tools for unraveling the molecular mechanisms underlying physiological and toxicological processes in various fields^7–9^. Systems toxicology approaches are also needed in nanotoxicology, in order to implement mechanism-based risk assessment. Transcriptomic assays probe the expression of the entire genome, identifying genes that are significantly up- or down-regulated under certain conditions, from the simultaneous measurement of tens of thousands of mRNA molecules. Metabolomics, which provides a snapshot of the physiological state by measuring small molecules and metabolites, usually reflects combined effects of multiple upstream factors, such as the transcriptome, proteome, and nutritional environment^9^. Metabolic profiling, thus, does not only permit biomarker identification, but it also provides mechanistic insights into chemical toxicity^10^. From this reason, the application of metabolomics to analyze the interactions of organisms with their environment, has grown considerably, as it can generate hypotheses involving non-targeted metabolomics of environmental stressors with unknown mode of action^11^. The integration of these OMICS technologies has the potential to reveal a more consistent view of cellular homeostasis and regulatory and signaling networks, compared to when used individually^9,12^.

The nematode *Caenorhabditis elegans* has been widely used as a model species because it offers many benefits: its genome has been sequenced with high fidelity, genetic variability is low because reproduction is primarily hermaphroditic, it is easily cultured in the laboratory, gene suppression can be easily administered by feeding, and there is a large collection of mutants already available^13–16^. Particular strengths of using *C. elegans* in the context of nanotoxicology lie in the potential to examine organismal uptake and distribution, due to its small size, transparency, and genetic power of single-celled systems in the context of the biological complexity of a metazoan with multiple well-developed organ systems^17^.

In this study, the phototoxicity of TiO_2_NPs was investigated on *C*. *elegans* exposed to TiO_2_NPs with and without UV activation (i.e. Control, TiO_2_NPs, UV, and UV + TiO_2_NPs). We mainly addressed two questions: i) how UV activation influences toxicity of TiO_2_NPs on *C. elegans*, using mortality, reproduction, and oxidative stress as endpoints; and ii) which are the molecular mechanisms of the interaction with TiO_2_NPs, with or without UV activation. To this end, we employed a systems toxicology approach, with global transcriptomics and metabolomics assays, followed by whole-genome network-based pathway analyses, with further experimental validation of the *in-silico-*derived hypothesis using functional genetics tools. Currently, the use of OMICS approaches to provide information on a chemical’s hazard and mode-of-action (MOA) is gaining acceptance in regulatory toxicology through the concept of adverse outcome pathways (AOP). In this context, the identified alterations of molecular pathways were interpreted with apical endpoint responses (i.e. reproduction), to elucidate the physiological meaning of these alterations. This approach will shed light on the potential of OMICS in the development of AOP.

## Results and Discussion

### Physicochemical characterization of TiO_2_NPs in K-media

The structure and size of TiO_2_NPs measured using TEM showed agglomerated nano-structure, formed the secondary agglomerates between the primary particles (SI Fig. S1A). In TEM images, the primary particle size was ca. 25–30 nm and the secondary particle size was ranged in 500–1000 nm. The aggregation behavior and colloidal stability of TiO_2_NPs were investigated at the concentrations used for toxicity tests (2, 5 and 10 mg/L), with and without UV, at under different time points (0, 6, 12 and 24-h; SI Fig. S1B, C). The particle size distribution of TiO_2_NPs, expressed as hydrodynamic diameters (HDDs) ranged from 1500 to 6000 nm. HDDs in K-media was increased with dispersion time and reached to 2000–6000 nm after 24-h, while the zeta potential in K-media ranged from 0 to −30 mV. Fluctuation of colloidal dispersion stability over time was similar for all treated concentrations regardless of UV activation. HDDs turned out to be between 2000 and 6000 nm, whereas zeta potential between 0 and −30 mV, regardless of UV activation (SI Fig. S1B, C). No clear effect dependent on concentration was observed in the DLS analysis. In addition, to confirm the photoactivation for visible-light, UV-vis diffuse reflectance spectrum was measured as shown in Fig. S2. TiO_2_NPs can absorb UV light owing to its wide bandgap (3.2 eV), whereas it has not continuous light absorption in 400–800 nm due to the intrinsic band-gap transition. Namely, TiO_2_NPs are likely to be only UV-light responsive photocatalyst, and thus it was not shown any photoactivation under visible-light condition.

### Effect of UV on worms‘ survival, reproduction and oxidative stress by TiO_2_NPs exposure

**Figure 1:**
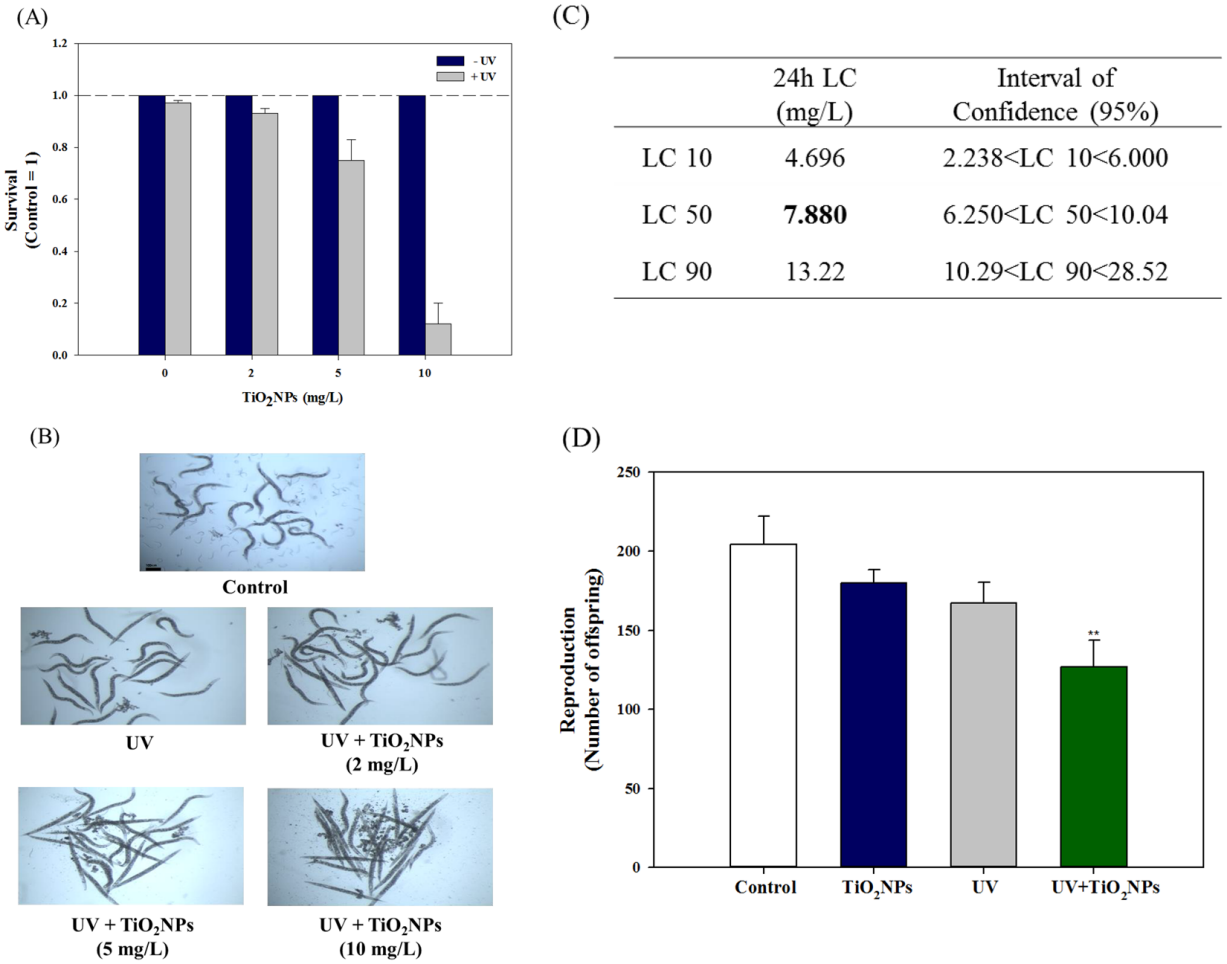
Lethal and reproductive toxicity of TiO_2_NPs in wildtype *C. elegans*. Survival (%, **A**), corresponding microscopic image (**B**), estimated 24-h LC50 (**C**), and reproductive toxicity (**D**). For lethal toxicity, *C. elegans* were exposed to 2, 5 and 10 mg/L of TiO_2_NPs via K-media for 24-h with/without UV activation (4-h with UV activation followed by 20-h recovery). Dead and live worms were counted for lethal toxicity and LC50 was estimated according to lethal response. For reproductive toxicity, young adult stage of *C. elegans* was exposed to TiO_2_NPs (5 mg/L) via K-media for 72-h according to four exposure scenarios (Control, TiO_2_NPs, UV, UV + TiO_2_NPs) and the number of offspring from each treatment was counted using COPAS. **Indicates statistically significant difference compared to Control (*p* < 0.01).

**Figure 2:**
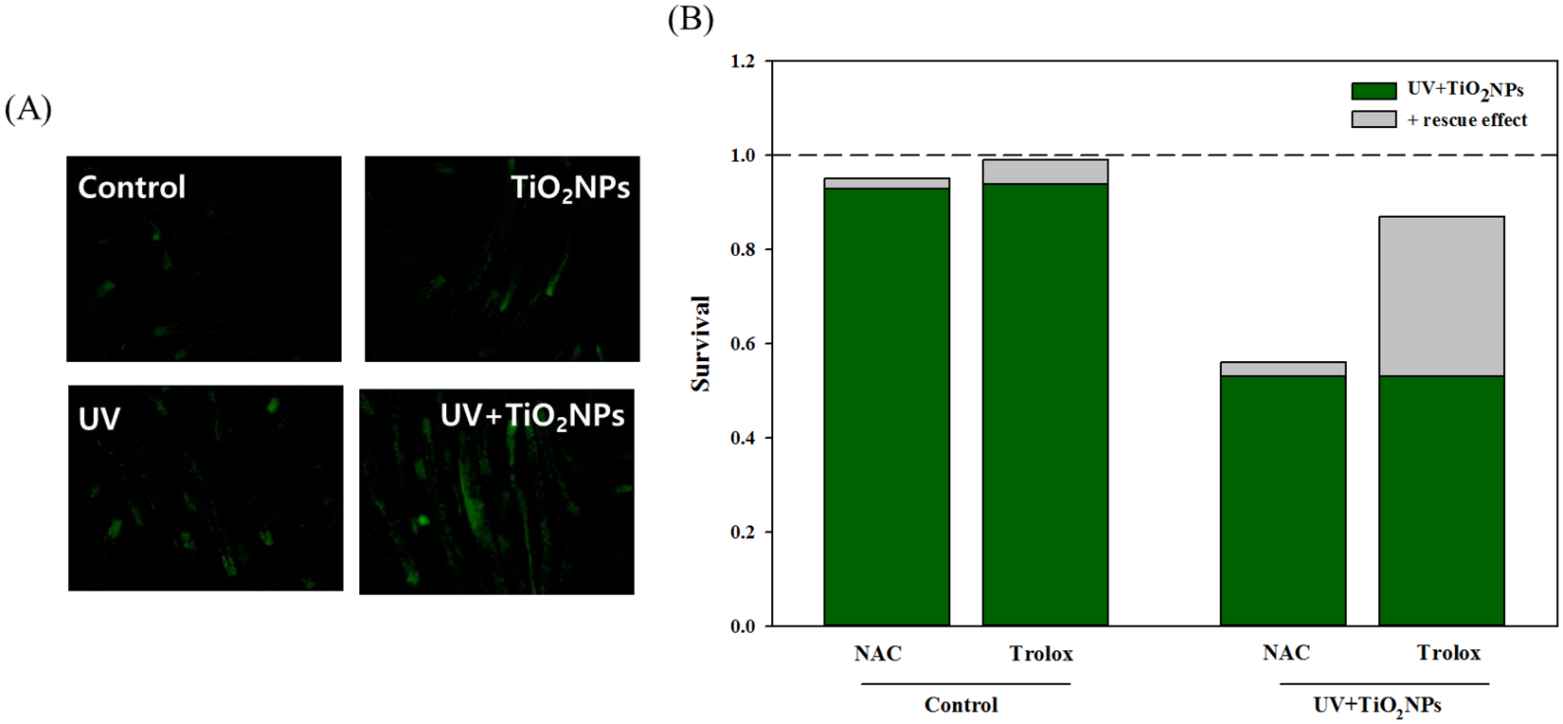
Oxidative stress of TiO_2_NPs in wildtype *C. elegans*. ROS formation assay using DCF-DA staining (**A**); pharmaceutical rescue assay using NAC and Trolox with/without UV + TiO_2_NPs (10 mg/L) (**B**).

To investigate the effect of UV on toxicity of TiO_2_NPs, mortality, reproduction and oxidative stress responses were examined in *C. elegans* exposed to TiO_2_NPs with and without UV irradiation (Figs 1 and 2). The 24-h mortality test revealed that, without UV activation, no mortality was observed up to 100 mg/L of TiO_2_NPs exposure, whereas 4-h UV activation followed by 20-h recovery led to significant toxicity in *C. elegans*. Under the UV-activated condition, significantly increased mortality was observed at 5 and 10 mg/L of TiO_2_NPs exposure, with almost 90% of mortality at 10 mg/L (Fig. 1A and B). LC10, 50 and 90 of TiO_2_NPs to *C. elegans* were thus estimated as 4.7, 7.9 and 13.2 mg/L, respectively (Fig. 1C). For further mechanistic study, LC10 was selected as exposure concentration (i.e. 5 mg/L). The effect of TiO_2_NPs on *C. elegans* was also investigated on worms‘ reproduction (Fig. 1D). Young adult *C. elegans* were exposed to 5 mg/L of TiO_2_NPs for 72-h with (4-h) and without UV, and the number of offspring was counted from each treatment (i.e. Control, TiO_2_NPs, UV, UV + TiO_2_NPs). UV-activated TiO_2_NPs exposure (UV + TiO_2_NPs) led to 37.9%, 29.5%, and 24.3% decrease in reproduction, which were significantly different from those of Control, TiO_2_NPs and UV alone exposure. The fact that UV + TiO_2_NPs exposure caused significant reproductive toxicity, while UV alone did not, suggests that UV activation was critical for the toxicity of TiO_2_NPs to *C. elegans*, and that reproductive toxicity was indeed related to TiO_2_NPs exposure. These results are consistent with the relevant literature, which has reported phototoxicity of TiO_2_NPs to various species^6^, showing clear evidence of increasing toxicity of TiO_2_NPs exposure together with UV, compared to TiO_2_NPs alone. Another study on the toxicity of TiO_2_NPs to L1 stage *C*. *elegans* for 24-h, without UV, reported a 24-h LC50 value of 80 mg/L^18^, much higher than ours. This discrepancy in LC50 values may have originated from differences in the state of exposure media and the physicochemical properties of TiO_2_NPs used. In the same study, the effect of TiO_2_NPs on reproduction was also evaluated for 5-d cultured L1 stage *C. elegans*, and it showed decreasing offspring per worm in a dose-dependent manner. Severe reactive oxygen species (ROS) formation by UV-activated TiO_2_NPs exposure has been previously reported in various *in vitro*- and *in vivo* models, such as freshwater green algae *(Pseudokirchneriella subcapitata)* or human keratinocyte HaCaT cells^19,20^. This effect was also found in our study, using DCF-DA staining and pharmaceutical rescue assays (Fig. 2). ROS formation, measured by fluorescence microscopy after staining worms with DCF-DA, increased more in the worms exposed to UV + TiO_2_NPs than in the Control and TiO_2_NPs treatments (Fig. 2A). Previously, the oxidative stress-induced toxicity of silver nanoparticles (AgNPs) was investigated by the antioxidant agent, N-acetylcysteine (NAC), which acts as a ROS scavenger and ion chelator, also by another antioxidant, Trolox, which only acts as a ROS scavenger. It was found that the toxicity of AgNPs was largely due to ROS and only partially due to dissolved Ag ions^21,22^. Here, toxicity rescue assays were conducted using the same pharmaceuticals, and no rescue was observed by NAC treatment, whereas significant rescue was observed with Trolox treatment, as expected (Fig. 2B). This result suggests that toxicity of UV + TiO_2_NPs is largely due to direct ROS formation. Generation of ROS was also found for TiO_2_NPs without UV activation, using toxicity tests on various mammalian cells^23^. From a more mechanism perspective, non-photoactivated induction of ROS by TiO_2_NPs via dissolved oxygen was reported in a recent study^24^. This implies that one possible toxicity mechanism of TiO_2_NPs might arise from ROS, and UV photoactivation can worsen this oxidative stress by increasing the level of ROS. Real exposure scenarios include distribution patterns of the materials of interest, physicochemical characteristics of environmental media, and other variables that can alter the activity or toxicity of nanomaterials. For instance, Clemente *et al* ^25^. performed toxicity tests on two aquatic system-dwelling organisms (*Daphnia similis, Artemia salina*), with minimal levels of UV light radiation. Their results underline that UV radiation enhances the toxicity of TiO_2_NPs, and that the levels of UV radiation in the real environmental water system are enough to increase TiO_2_NPs toxicity. Additionally, Ma *et al* ^26^. reported that the spectrum of solar radiation can be a relevant factor in the phototoxicity of TiO_2_NPs. By using different spectrum filters, it was found that ROS generation by TiO_2_NPs was mainly activated by UV-A (320–400 nm) radiation, rather than by UV-B (280–320 nm) radiation. This result implies that in realistic environmental situations, considering the spectrum of UV irradiation is needed in order to manage the safe usage of nanomaterials. Nevertheless, this result seems to differ from ours, because we also found ROS generation in a UV-B irradiation situation, so further studies are needed to understand the degree of photoactivation of TiO_2_NPs depending on UV wavelength and its influence on TiO_2_NPs toxicity.

### Transcriptomics

**Figure 3:**
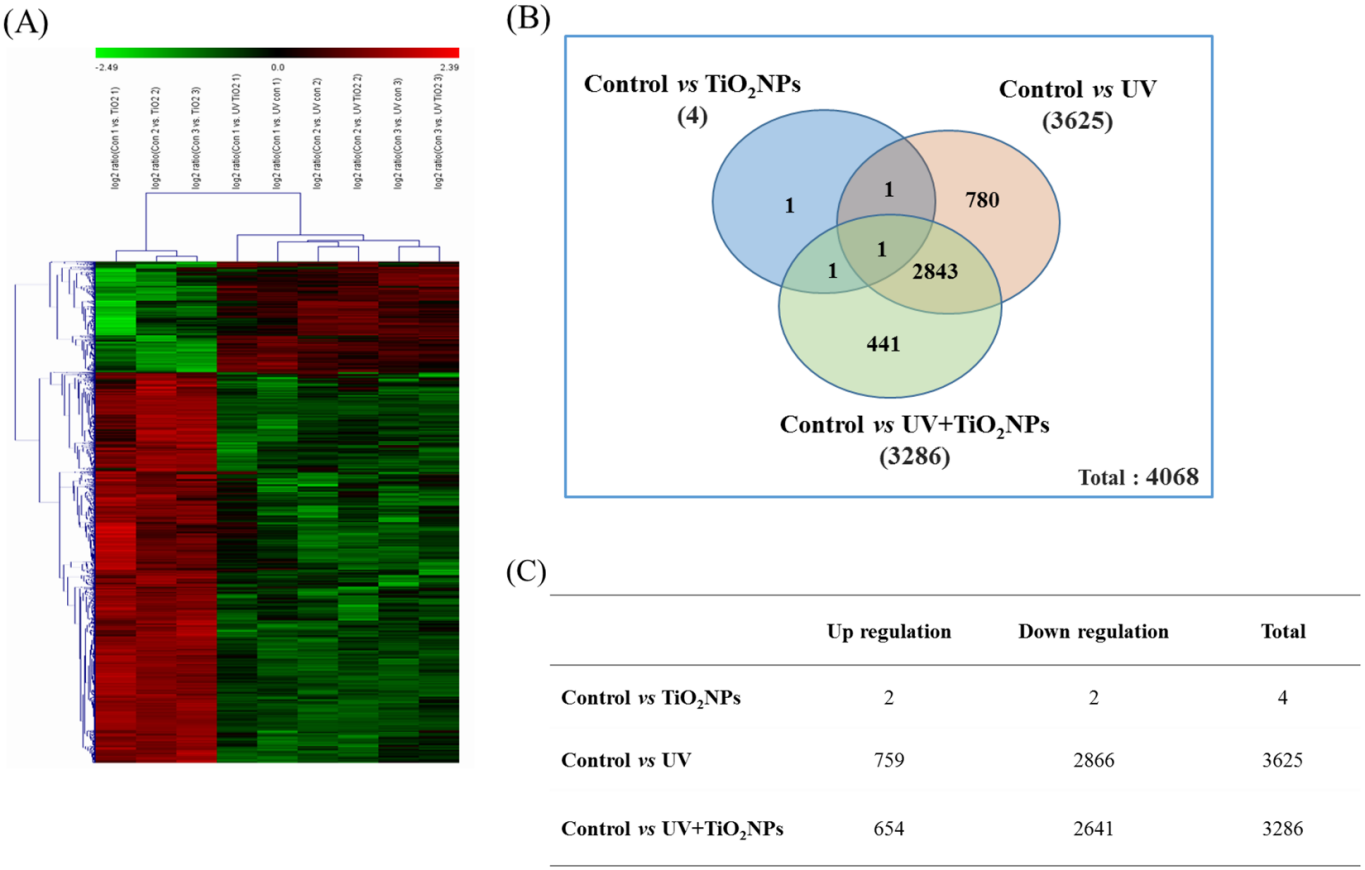
Differentially expressed genes (DEGs) by TiO_2_NPs, UV, UV + TiO_2_NPs exposure in wildtype *C. elegans* based on microarray experiment. Heat map from the hierarchical clustering of DEGs (**A**), Venn diagram on the overlap DEGs (**B**) and up- and down regulated DEGs on exposure to TiO_2_NPs, UV, UV + TiO_2_NPs (**C**). The number of expressed genes determined by microarray analysis are displaying 2-fold changes in expression.

As we found significant toxicity by UV-activated TiO_2_NPs in *C. elegans* (i.e. reproduction failure and increased mortality, Fig. 1), with involvement of oxidative stress (Fig. 2), we performed global microarray-based gene expression assays to explore the underlying mechanisms of UV-activated TiO_2_NPs in *C. elegans*. Young adult *C. elegans* were exposed to 5 mg/L of TiO_2_NPs for 24-h with and without 4-h UV activation and, together with unexposed worms, were harvested for microarray analysis. The heat map of hierarchical clustering of differentially expressed genes (DEGs) clearly shows UV and UV + TiO_2_NPs groups clustered by distinct expression pattern with respect to the TiO_2_NPs group (Fig. 3A). Microarray analysis revealed only 4 DEGs by TiO_2_NPs alone, compared to 3,625 DEGs by UV alone and 3,286 DEGs by UV-activated TiO_2_NPs exposure (Fig. 3B). From the comparison of DEGs from UV and UV + TiO_2_NPs, 2,844 DEGs were common, 780 genes were specific to UV, and 441 genes were specific to UV + TiO_2_NPs. More genes were down-regulated than up-regulated by UV ± TiO_2_NPs; 2,866 and 2,641 genes were down-regulated by UV alone and TiO_2_NPs, respectively, while 759 and 654 genes were up-regulated (Fig. 3C). From a recently conducted microarray study of *Pseudomonas aeruginosa* PA01 bacterium cells using TiO_2_-coated ethylene-vinyl alcohol (EVOH) particles, under irradiation of UV-B light and low doses of UV and TiO_2_^27^, UV activation induced lower expressions in signaling, regulation and cell wall structure when compared to the UV-irradiated EVOH treatment. Also with the comparison between UV-irradiated TiO_2_-coated EVOH and Control (UV-irradiated EVOH), the total number of DEGs was 151 down-regulated and 165 up-regulated. Also with zebrafish embryos exposed to 20 ng/l of TiO_2_NPs, the total number of DEGs, respectively to Control, was 360 and 198 up-regulated and 162 down-regulated^28^. Our results, compared with these other microarray studies, there is consistency in pattern of up- or down-regulation when exposed to TiO_2_NPs, but revealed a smaller number of DEGs from sole TiO_2_NPs exposure. Apart from differences in the organisms used, this difference can be caused by differences in the experimental designs and statistical analyses.

### Pathway analysis of DEGs from transcriptomics

Microarray assays reflected the effect of UV activation on the exposure to TiO_2_NPs, so this would correlate to the organism-level toxicity we observed. To understand the mechanisms of phototoxicity of TiO_2_NPs, whole-organism network-based pathway analyses were conducted (Table 1). Significantly altered pathways were not found by exposure to TiO_2_NPs alone, whereas various stress response pathways, such as xenobiotic metabolism, oxidative stress, DNA repair, and stress signaling pathways were found to be significantly altered by UV-alone exposure. Alteration on various DNA repair pathways (i.e. base excision repair, nuclear excision repair, mismatch repair, homologous recombination, non-homologous end-joining) was highly significant with UV exposure. Interestingly, most significantly altered pathways by exposure to UV alone, including the DNA repair pathways, were also found to be differentially altered with UV + TiO_2_NPs, which suggests that few effects were specific to UV + TiO_2_NPs. Moreover, when comparing UV *vs* control, UV + TiO_2_NPs *vs* control, and UV + TiO_2_NPs *vs* TiO_2_NPs, many pathways were found to be significantly altered in these three comparisons, which again suggest that they were mainly caused by UV exposure. On the other hand, TiO_2_NPs-specific pathways, evidenced by the comparison of UV *vs* UV + TiO_2_NPs, turned out to be the JAK/STAT and xenobiotic metabolism pathways (i.e. cytochrome P450). Among them, in an attempt to understand the mechanisms of the reproductive toxicity observed with UV + TiO_2_NPs exposure (Fig. 1D), we focused on the JAK/STAT pathway for further analysis, as this pathway is known to control many biological processes during embryonic and larval development^29^. To further explore the biological meaning of the observed transcriptional alteration, we identified pathways related to metabolites based on DEGs from transcriptomics (SI Table S3). Similar to the transcriptomics results, UV + TiO_2_NPs led to the alteration of various metabolite-related pathways. when compared to Control and to TiO_2_NPs, whereas when compared to UV, lesser pathways were differentially affected, which again suggests few TiO_2_NPs-specific effects, Taurine metabolism was found among the pathways specifically affected by TiO_2_NPs. This pathway has been previously reported to be involved in male and female reproduction in mammalian models^30,31^. This indicated possible metabolites involved in the observed reproductive toxicity of TiO_2_NPs, which led us to perform metabolic profiling at the same exposure settings as the transcriptomics assays.

**Table 1:** List of pathways detected by the network analysis conducted on differentially expressed genes.

Pathway ID	Pathway description	*p*-value			
		TiO_2_NPs vs Control	UV vs Control	UV+TiO_2_NPs vs TiO_2_NPs	UV+TiO_2_NPs vs UV
cel00480	Glutathione metabolism	8.88E-01	2.22E-04*	2.10E-04*	7.76E-02
cel00980	Metabolism of xenobiotics by cytochrome P450	8.88E-01	2.22E-04*	2.10E-04*	4.46E-02*
cel00982	Drug metabolism - cytochrome P450	8.88E-01	2.22E-04*	2.10E-04*	4.76E-02*
cel00983	Drug metabolism - other enzymes	8.88E-01	2.22E-04*	2.10E-04*	4.76E-02*
cel03410	Base excision repair	8.88E-01	2.22E-04*	2.10E-04*	3.62E-01
cel03420	Nucleotide excision repair	8.88E-01	2.22E-04*	2.10E-04*	4.31E-01
cel03430	Mismatch repair	8.88E-01	2.22E-04*	2.10E-04*	3.62E-01
cel03440	Homologous recombination	8.88E-01	2.22E-04*	2.10E-04*	2.89E-01
cel03450	Non-homologous end-joining	8.88E-01	2.22E-04*	2.10E-04*	4.57E-01
cel03460	Fanconi anemia pathway	8.88E-01	2.22E-04*	2.10E-04*	3.93E-01
cel04010	MAPK signaling pathway	8.88E-01	2.22E-04*	2.10E-04*	3.93E-01
cel04012	ErbB signaling pathway	8.88E-01	2.22E-04*	2.10E-04*	2.60E-01
cel04020	Calcium signaling pathway	8.88E-01	2.22E-04*	2.10E-04*	2.31E-01
cel04070	Phosphatidylinositol signaling system	8.88E-01	2.22E-04*	2.10E-04*	2.89E-01
cel04080	Neuroactive ligand-receptor interaction	8.88E-01	2.22E-04*	2.10E-04*	3.27E-01
cel04140	Regulation of autophagy	8.88E-01	2.22E-04*	2.10E-04*	4.85E-01
cel04141	Protein processing in endoplasmic reticulum	8.88E-01	2.22E-04*	2.10E-04*	5.12E-01
cel04142	Lysosome	8.88E-01	2.22E-04*	2.10E-04*	1.09E-01
cel04144	Endocytosis	8.88E-01	2.22E-04*	2.10E-04*	4.85E-01
cel04145	Phagosome	8.88E-01	2.22E-04*	2.10E-04*	2.89E-01
cel04150	mTOR signaling pathway	8.88E-01	2.22E-04*	2.10E-04*	3.93E-01
cel04310	Wnt signaling pathway	8.88E-01	2.22E-04*	2.10E-04*	2.89E-01
cel04350	TGF-beta signaling pathway	8.88E-01	2.22E-04*	2.10E-04*	3.93E-01
cel04512	ECM-receptor interaction	8.88E-01	2.22E-04*	4.20E-04*	1.46E-01
cel04630	Jak-STAT signaling pathway	8.88E-01	2.22E-04*	2.10E-04*	4.46E-02*
cel04330	Notch signaling pathway	9.02E-01	6.26E-03*	2.10E-04*	5.46E-01
cel04340	Hedgehog signaling pathway	9.09E-01	2.22E-04*	2.10E-04*	1.74E-01

(*Indicates significant difference at the *p* < 0.05 pathway for each comparison).

### Global metabolomics and pathway analysis

To elucidate whether TiO_2_NPs-induced metabolic alterations might lead to reproductive toxicity, non-targeted global metabolomics assays were performed using ^1^H-NMR (SI Fig. S3). Based on global metabolite patterns, four treatment groups (Control, TiO_2_NPs, UV, UV + TiO_2_NPs) were first compared together using OPLS-DA. Score plots indicated that Control and TiO_2_NPs treatments, on one hand, and UV and UV + TiO_2_NPs treatments, on the other, clustered together (SI Fig. S4). Further comparison between the UV and UV + TiO_2_NPs treatments revealed distinct clusters separating both groups (SI Fig. S4B), suggesting an effect specific to TiO_2_NPs in the exposure to UV + TiO_2_NPs, compared to UV alone. Metabolites differentially affected by TiO_2_NPs, with respect to Control, were identified by an ANOVA test. Significantly altered metabolites in *C. elegans* treated with TiO_2_NPs, with and without UV activation, are presented in SI Table S4. Significant accumulation of succinate, aspartate and acetate, as well as significant depletion of pyruvate, alanine, acetamide, betaine, choline, and tyrosine were observed in the UV + TiO_2_NPs treatment compared to UV alone, which suggests an alteration on energy metabolism, especially evidenced by the accumulation of succinate and depletion of pyruvate. To further understand the biological meaning of these significant changes in metabolite concentration, pathways were analyzed with MetaboAnalyst 3.0 software, using significantly altered metabolites (*p < *0.05) (SI Fig. S5). Among others, methane and tryptophan metabolism pathways were up-regulated, while glycerol phospholipid, cyanoamino acid, glycine, serine, and threonine metabolism pathways were down-regulated by UV + TiO_2_NPs exposure compared to Control. Metabolic pathways specifically related to TiO_2_NPs phototoxicity were suggested by the comparison between UV + TiO_2_NPs and UV treatments, which revealed up-regulation of alanine, aspartate, glutamate, and sulfur metabolism pathways, together with down-regulation of cyanoamino acid, glycine, serine, threonine, aminoacyl-tRNA, and tyrosine metabolism pathways (SI Fig. S5B). Metabolomics assays have been previously used to investigate the response of *C. elegans* to environmental stressors^15,16,32–34^. Among them, cadmium toxicity was investigated using ^1^H-NMR, LC–MS, and metabolomic analysis, which resulted in the proposal of phytochelatin-bound cadmium as a possible detoxification mechanism in cadmium-exposed *C. elegans* ^32^. Here, we found alteration of various amino acid metabolism pathways by TiO_2_NPs, such as alanine, aspartate, glutamate, glycine, serine, and threonine metabolism, many of which seem to be directly or indirectly related to reproductive toxicity. As amino acids are key regulators of metabolism, growth, development, immune response, and health^35^, effects on amino acids could be translated to higher level effects, such as reproduction. Indeed, metabolomics has recently been proposed as a potential approach for investigating human reproductive disorders. High correlation was found between the clinical parameter (sperm concentration) and the metabolite profiles generated from serum of the study participants, Danish young men^36^. In another study, changes in functional amino acid metabolism, in particular, altered the reproductive performance in males and females, as well as their offspring, through the abundance and activity of intestinal bacteria^37^. More closely related with our work, Ratnasekhar *et al* ^38^. reported that differential metabolic profiles in *C. elegans* exposed to TiO_2_ (NPs or bulk particle) were directly related to effects on reproduction.

### Functional analysis of JAK/STAT and TGF-ß pathways

UV-activated TiO_2_NPs led to significant reproductive toxicity in *C. elegans* (Fig. 1D), which was also supported by the metabolic profiling (SI Table S4 and SI Fig. S5), while the JAK/STAT pathway was significantly altered by TiO_2_NPs, as evidenced by the pathway analyses of transcriptomics results (Table 1). The JAK/STAT pathway is known to regulate development and reproduction in many organisms. Although it is not fully conserved in *C. elegans*, several STAT proteins has been found in *C. elegans*, with functional studies yet to be reported^39^. From previous studies conducted on endothelial cells, activation of the JAK2/STAT3 signaling pathway was found when oxidative stress was introduced, whereas inhibition of the JAK2/STAT3 pathway resulted in decreased oxidative stress injury^40,41^. Following this, the functional role of the JAK/STAT pathway in oxidative stress related to toxicity was reported in an experiment with mouse exposed to far-infrared radiation (FIR)^42^. Consistent with the role of glutathione peroxidases (GPx) as antioxidant enzymes^43^, exposure to FIR induced inhibition of the JAK2/STAT3 signaling pathway, in correlation with hyper-activation of GPx-1. Although these results were obtained with other model organisms and different exposure scenarios, all of them share a common toxicity mechanism, oxidative stress. Moreover, our results also highlight the role of glutathione metabolism and the JAK/STAT signaling pathway. The oxidative stress induced by UV-activated TiO_2_NPs exposure, evidenced by the pharmaceutical rescue assay (Fig. 2), and the inhibition of glutathione metabolism detected by metabolomics (SI Fig. S5), jointly support the hypothesis that the toxicity specific to TiO_2_NPs also results from down-regulation of glutathione, as a result from JAK/STAT signaling pathway reacting to oxidative stress.

**Figure 4:**
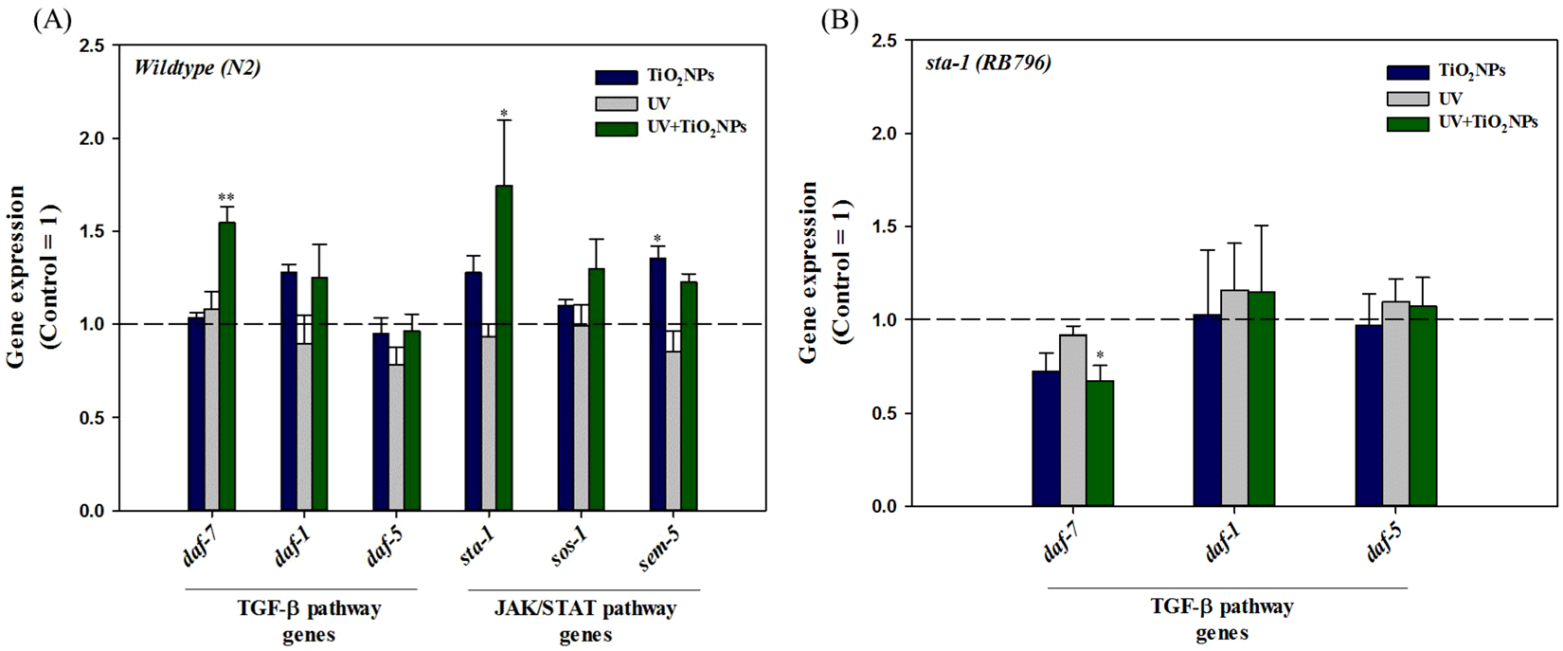
The expression of TGF-ß pathway genes (*daf-7, daf-1 and daf-5)* and JAK/STAT pathway genes (*sta-1, sos-1 and sem-5*) in wildtype (**A**), and TGF-ß pathway genes (*daf-7, daf-1 and daf-5*) in *sta-1* mutant (**B**). Wildtype and *sta-1* mutant *C. elegans* were exposed to TiO_2_NPs, UV and UV + TiO_2_NPs and gene expression was analyzed using qRT-PCR. The results were expressed as the mean value compared to Control (Control = 1, n = 3; mean standard error of the mean; two-tailed t-test, *p < 0.05; **p < 0.01).

**Figure 5:**
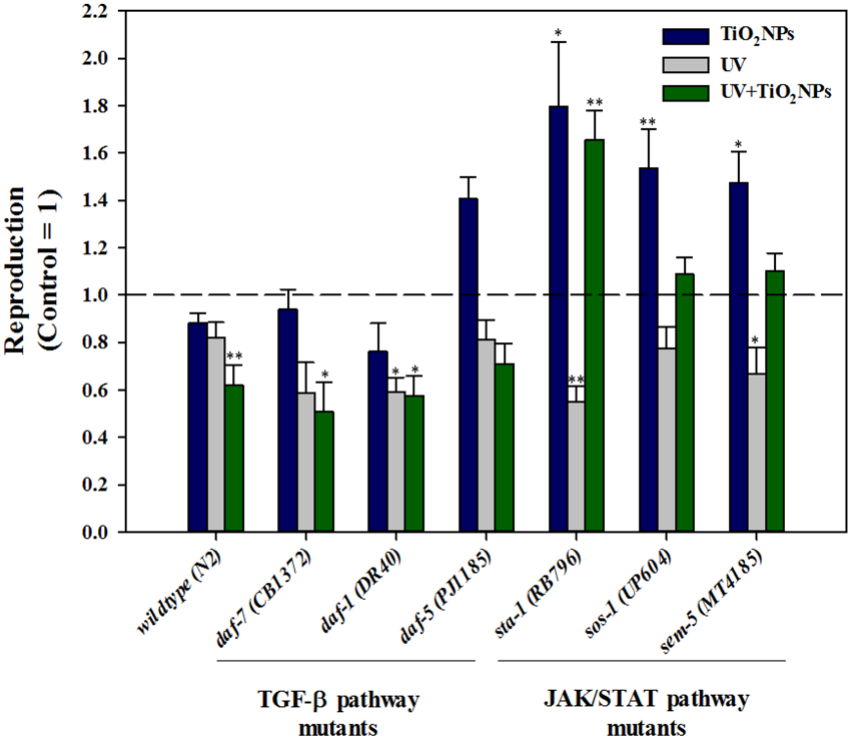
Reproduction of mutants of genes in TGF-ß pathway and JAK/STAT pathways on *C. elegans* exposed to TiO_2_NPs, UV and UV + TiO_2_NPs. Reproduction was investigated by counting the number of offspring 72-h after exposure to TiO_2_NPs, UV and UV + TiO_2_NPs on TGF-ß pathway genes mutants (*daf-7 (CB1372), daf-1 (DR40) and daf-5 (PJ1185*)) and JAK/STAT pathway genes mutants (*sta-1 (RB796), sos-1 (UP604) and sem-5 (MT4185)*) *C. elegans*. The results were expressed as the mean value compared to Control for each mutant. (Control = 1, n = 8; mean standard error of the mean; two-tailed t-test, *p < 0.05; **p < 0.01).

**Figure 6:**
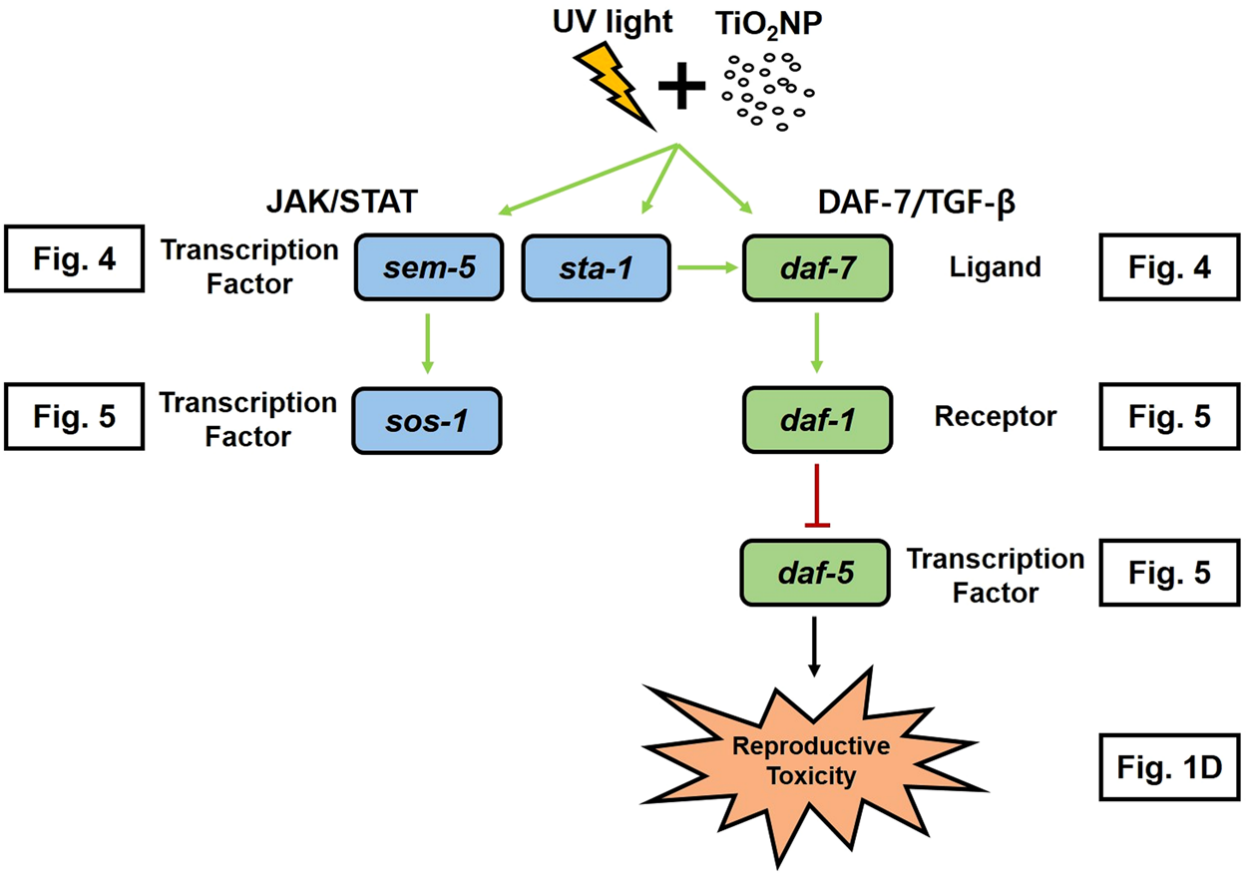
Proposed model of JAK/STAT and DAF-7/TGF-ß crosstalk pathway mediated reproductive toxicity in UV-activated TiO_2_NPs exposed *C. elegans*.

Therefore, to experimentally confirm this pathway as a mechanism of phototoxicity of TiO_2_NPs, a functional genetic study was conducted, focused on the JAK/STAT pathway. This pathway is also known to crosstalk with TGF-ß and DAF-16/FOXO pathways^44^, both of which are key regulators of development, reproduction, life span and stress response in *C. elegans* ^45–47^. Thus, we investigated the TGF-ß pathway along with the JAK/STAT pathway as potential key pathways in TiO_2_NPs phototoxicity. Thus, the reduction in reproduction observed in the UV + TiO_2_NPs treatment might be related to the alteration of these pathways. The same exposure scenarios (i.e. Control, TiO_2_NPs, UV, UV + TiO_2_NPs) as in the basal reproductive toxicity (Fig. 1D) and OMICS assays (Fig. 3 and SI Fig. S3) were also applied for the gene expression and functional genetic studies. Expression of genes *sta-1, sos-1, sem-5* (JAK/STAT pathway) and *daf-7, daf-1, daf-5* (TGF-ß pathway) was investigated (Fig. 4A). Increased expression of *sem-5* gene was observed with TiO_2_NPs exposure, whereas increased expression of *sta-1* and *daf-7* was observed with UV + TiO_2_NPs. This suggests again the involvement of JAK/STAT and TGF-ß pathways in the reproductive failure induced by UV-activated TiO_2_NPs. To pursue this hypothesis, worms‘ reproduction with TiO_2_NPs exposure was also examined using loss-of-function mutants (i.e. *sta-1, sos-1* and *sem-5* for JAK/STAT; *daf-7, daf-1* and *daf-5* for TGF-ß, Fig. 5). Effects specific to TiO_2_NPs were investigated by comparing UV + TiO_2_NPs to UV. UV-induced reproductive toxicity was rescued in UV + TiO_2_NPs condition by JAK/STAT mutants *(sta-1, sos-1* and *sem-5*), whereas not by TGF-ß mutants, confirming the role of the JAK/STAT pathway in TiO_2_NPs phototoxicity. And UV-specific effects were investigated by comparing UV + TiO_2_NPs to TiO_2_NPs. No difference was found in the response of JAK/STAT mutants, whereas toxicity of TiO_2_NPs was exacerbated by UV radiation in TGF-ß mutants, indicating a functional role of the TGF-ß pathway in UV toxicity. The DAF-7/TGF-ß pathway in *C. elegans* is known to interpret environmental signals relayed through neurons, and it inhibits dauer formation during reproductive developmental growth. Thus, activation of the TGF-ß ligand *daf-7* is expected to promote reproductive growth^48,49^. Our results show that UV activated TiO_2_NPs lead to a significant decrease in reproduction (Fig. 1D) and to a significant increase in the expression of the *daf-7* gene (Fig. 4A). Toxicity test with loss-of-function mutants revealed that reproductive toxicity of the *daf-7* mutant under UV + TiO_2_NPs exposure condition was exacerbated compared to that of wildtype (Fig. 5). These collectively suggest that the role of the TGF-ß pathway in *C. elegans* reproductive development also holds true in the stressful condition of photoactivated TiO_2_NPs exposure (UV + TiO_2_NPs). Next, we investigated the crosstalk between the JAK/STAT and TGF-ß pathways on UV ± TiO_2_NPs exposure, by examining the expression of TGF-ß pathway genes in the *sta-1* mutant (Fig. 4B). Previously, crosstalk relationship of JAK/STAT and TGF-ß pathways in repressing dauer formation was reported^48^ by the observation that loss of STA-1 resulted in enhanced TGF-ß target gene expression. In our case, increased expression of the *daf-7* gene with UV + TiO_2_NPs did not occur in the *sta-1* mutant (Fig. 4B), which indicates a possible special crosstalk between the JAK/STAT and TGF-ß pathways in TiO_2_NPs phototoxicity. Gene expression and functional mutant assays collectively suggest that, in *C. elegans*, the JAK/STAT pathway seems to cooperate with DAF-7/TGF-ß signaling in maintaining reproductive growth with UV-activated TiO_2_NPs (Fig. 6).

Overall, our results show that UV and UV-activated TiO_2_NPs induced significant toxicity to *C. elegans*, and the phototoxicity of TiO_2_NPs induced up-regulation of JAK/STAT pathway target gene *sta-1*, and TGF-ß pathway target gene *daf-7*, in accordance with their role in reproductive growth. Overall, possible relationship between *sta-1* and *daf-7* gene and crosstalk partner for STA-1 with DAF-7/TGF- ß pathway were suggested with different signaling pathways involved in TiO_2_NPs-specific and UV-specific phototoxicity.

## Conclusion

As exposure scenarios greatly alter the risk of nanoparticles, conducting nanotoxicity tests that reflect real environmental conditions is gaining importance, in order to develop appropriate risk assessment and regulation, and to guide the proper use of nanomaterials. In this context, thorough investigation of the toxicity mechanisms of TiO_2_NPs under real exposure scenarios is needed for the safe use of TiO_2_NPs. Currently, the use of OMICS approaches to gather information on a chemical’s hazard and MOA is gaining acceptance in regulatory toxicology through the concept of AOP. This study identified the JAK/STAT and TGF-ß pathways as molecular mechanisms involved in TiO_2_NPs-induced reproductive toxicity. Further studies are needed to identify molecular initiating events (MIE), key events (KE), and their causal relationship with reproductive toxicity, to establish the AOP of reproductive toxicity *via* the JAK/STAT and TGF-ß pathways. We believe that our study will contribute to a better understanding of the mechanisms of TiO_2_NPs phototoxicity. It also presents a promising example of the elucidation of regulatory signaling cascades induced by nanomaterials, by means of integrated single-gene (RT-qPCR, loss-of-function mutants) and OMICS (transcriptomics and metabolomics) assays, together with network-based whole-organism pathway analysis.

## Materials and Methods

### *C. elegans* strains and maintenance

*C. elegans* were grown in Petri dishes on nematode growth medium (NGM) and fed OP50 strain *Escherichia coli* according to a standard protocol^50^. Worms were incubated at 20 °C, and age-synchronized young adults (3 days after the age-synchronizing procedure) were used in 72-h reproduction experiments. *Wildtype (N2)* and *daf-1 (DR40)*, *daf-5 (PJ1185), daf-7 (CB1372), sta-1 (RB796), sos-1 (UP604), sem-5 (MT4185)* mutants were provided by the Caenorhabditis Genetics Center (www.CGC.org) at the University of Minnesota (Minneapolis, MN, USA). A list of the mutant strains and descriptions are presented in Supplementary Information (SI) Table S1.

### Preparation and characterization of TiO_2_NPs

TiO_2_NPs powder (Degussa P-25, purity > 99.5%) were purchased from Evonik (Germany). It is a standard material in the field of photocatalytic reactions, contains anatase and rutile phases in a ratio of about 8: 2, having average primary particle size 21 nm. To investigate the size and shape of the nanoparticles, 2 L of a particle suspension from the test medium was dried on a 400-mesh carbon-coated copper grid and imaged using a JEM 1010 TEM (JEOL, Japan) at 40–100 Kv (SI Fig. S1A). The particle size distribution of TiO_2_NPs in K-media (0.032 M KCl and 0.051 M NaCl) was characterized using hydrodynamic diameters (HDDs) and zeta potential, which were measured by dynamic light scattering spectroscopy (DLS, ELS-Z Photal, Japan) (SI Fig. S1B , C). The stock of TiO_2_NPs was prepared in K-media by sonicating for 30 min (Branson-5210 sonicator, Branson). From stock solutions (100 mg/L), experimental concentrations of TiO_2_NPs were obtained by dilution in K-media.

### Lethality test and pharmaceutical rescue assay

Lethality tests were performed on the young adult stage *wildtype (N2) C. elegans* after 24-h of exposure to 0, 2, 5, 10 mg/L of TiO_2_NPs in K-media without food. All groups of worms were maintained at 20 °C incubator, with UV-exposed groups under the UV-B lamp for the first 4-h. A UV-B lamp (G8T5E, Sankyo Denki, Japan) was installed in the 20 °C incubator, with height adjusted to meet the desirable UV intensity, using a UV meter (Model 850009, Super Scientific, US). UV exposure duration and intensity (4-h, 0.642 mW/cm²) was determined based on pre-test results which *wildtype (N2) C. elegans* was exposed to UV in this manner in K-media and did not show lethal effect. After 24-h, the number of live and dead worms was determined by visual inspection, probing with a platinum wire under a dissecting microscope. LC50 were derived through Probit analysis. To verify the effect of oxidative stress induced by TiO_2_NPs under UV light to worm’s survival rate, *wildtype (N2)* worms were pretreated with 10 mg/L Trolox and N-acetylcysteine (NAC), followed by treatment of 0 and 10 mg/L TiO_2_NPs, respectively. After 24-h exposure, the worms were checked for survival. All experiments were conducted on three biological replicates. Detailed exposure conditions for each endpoint are described in Table S2.

### Reproduction assay

Control and TiO_2_NPs groups were incubated at 20 °C for 72-h. UV, and UV + TiO_2_NPs groups were incubated at 20 °C under UV exposure for 4-h, and thereafter transferred to the same incubator as the Control and TiO_2_NPs group for the next 68-h. A UV-B lamp was installed in the 20 °C incubator, with height adjusted to meet the desirable UV intensity, using a UV meter. UV exposure duration and intensity (4-h, 0.642 mW/cm²) was determined based on pre-test results, where *wildtype (N2) C. elegans* was exposed to UV in this manner in K-media and did not show lethal effect. Reproduction test was conducted on *wildtype (N2)* as well as mutant strains by measuring the number of offspring from one young-adult after 72-h exposure in the four scenarios mentioned above, by using COPAS (complex object parametric analysis and sorting)-SELECT™.

### ROS formation assay

To detect the internal levels of reactive oxygen species (ROS), *wildtype (N2) C. elegans* were exposed to 0 and 10 mg/L of TiO_2_NPs, with or without UV exposure, for 24-h, then transferred to 0.5 ml of S buffer, containing 30 mM 2,7-dichlorofluoroscein diacetate (DCFH-DA), a well-established compound used for detecting ROS^51^. DCFH-DA reacts with ROS, and abundance of ROS can be observed by fluorescent intensity. The fluorescence was observed with a Leica DM IL microscope, with images obtained using a Leica DCF 420 C camera. Levamisole (2 Mm, Sigma-Aldrich) was applied to *C. elegans*, and pictures of the live worms were taken after 4- and 24-h.

### Transcriptomics and pathway analysis

Age synchronized young adult worms were pooled from the four exposure conditions. The total RNA from each group was prepared according to the standard protocol of the RNeasy Mini kit (Qiagen, Hilden, Germany). Five l g aliquots of each total RNA product were used for reverse and *in vitro* transcription followed by application to a GeneChip *C. elegans* Genome Array (Affymetrix, Santa Clara, CA, USA), which contained 22,500 probe sets against 22,150 unique *C. elegan*s transcripts. After the final wash & staining step, GeneChips were scanned using Affymetrix Model 3000 G7 scanner and the image data was extracted through Affymetrix Command Console software v.1.1. Expression data were generated by Affymetrix Expression Console software v 1.1. Microarrays were obtained from three biological replicates of each of the four treatment groups, thus 12 GeneChips were used in total. Pathways were analyzed using a gene-pathway network, with an approach similar to the one employed in previous studies^52,53^. The gene-pathway network was built by considering genes as nodes, connected by links consisting of shared pathways, i.e. pathways to which both genes were annotated. By construction, paths in this network represent possible routes of interaction between pathways, mediated by common genes. The advantage of this approach, compared to more standard pathway analyses based on considering each pathway in isolation, is the potential to identify crosstalk between pathways. KEGG pathways, genes, and annotations to *C. elegans* ^54^ were used, resulting in a network with 1,922 nodes (genes) connected by 78,489 links (shared pathways), from a set of 119 pathways. All shortest paths between any two nodes in the network were identified with Dijkstra’s algorithm^55^. Differentially expressed genes (DEG) from the several comparisons between experimental conditions were selected, using a compound threshold of p-value < 0.01 and fold change > 2, following recommendations for microarray studies^56^. Each gene and pathway was assigned a network score, representing differential expression in the context of the gene-pathway network. Scores had an initial value equal to zero. Each shortest path in the network whose nodes were all DEG added one to the score of the nodes and links (genes and pathways) that composed it. The statistical significance of the network scores was evaluated by comparing with an empirical null distribution, obtained by calculating the network scores of 1,000 random sets of DEG, equal in size to each actual set of DEG mapped to the network.

### NMR based metabolomics and pathway analysis

The NMR-based metabolomics were performed in *C. elegans*, obtained from the four exposure conditions (Control, TiO_2_NPs, UV, UV + TiO_2_NPs). Each sample was collected and washed with 1X-K media buffer containing deuterium oxide (D_2_O). All samples were spun down and the supernatant of each sample was eliminated. All samples were stored at −80 °C until analysis. 35 μL of sample and 10 μL of D_2_O containing 2 mM TSP-d_4_ (3-(trimethylsilyl) propionic-2,2,3,3-d_4_ acid sodium salt) was added to NMR nano tube. The samples was loaded into a 4 mm nano zirconium rotor. The total volume was adjusted to 45 µL with deuterium oxide to provide filed lock, and the samples also contained 2 mM TSP-d_4_ as a reference. A lid was capped as a closure of the rotor and marked at the rotor for the monitoring of spinning speed^29,30^. ^1^H-NMR experiments were carried out on an Agilent 600 spectrometer (Agilent Technologies, CA, USA) operating at 600.17 MHz. All instruments were equipped with a gH(X) nano probe. All data were collected at a spinning rate of 2,000 Hz and the spectra were checked between the water peak and the sideband, which coincide with the spin rate. Spectra were recorded at 299.1 K with a spectral width of 9,600 Hz, an acquisition time of 3.0 s, a relaxation delay of 1.0 s, and 128 scans. Measurement of 1D proton NMR spectra acquired with CPMG (Carr-Purcell-Meiboom-Gill) pulse sequence to suppress water signal and macromolecules. The total acquisition time was 9 min 56 sec. In all spectra, 0.2 Hz line broadening was applied prior to FT using Vnmrj (version 3.1 Agilent Technologies, CA, USA). All data were FT and calibrated to TSP-d_4_ as 0.00 ppm using Chenomx NMR suite 7.1 professional (Chenomx Inc., Edmonton, Canada). All spectra were processed and assigned by Chenomx NMR suite 7.1 professional and the Chenomx 600 MHz library database. All data were converted to the frequency domain and corrected for phase and baseline, and then the TSP-d_4_ singlet peak was adjusted to 0.00 ppm. Multivariative statistical analyses were established with all samples using SIMCA-P + 12.0.1 software package (Umetrics, Umeå, Sweden). Normalization of the total area of the spectrum was applied to each sample data set in order to minimize the effects of variable concentration among different samples. Orthogonal projection to latent structures-discriminant analysis (OPLS-DA) analysis was performed to differentiate between the control and treated groups. Metabolomics enrichment pathway analysis were performed by using MetaboAnalyst software 3.0 (a web service for metabolomics data analysis)^57^ with the metabolites that displayed > 1.5 fold changes and significantly distinct (*p* < 0.05) than control. As with microarrays, metabolomics were individually performed on three biological replicates at each of four treatment group, thus 12 metabolic profiles were used in total.

### Quantitative real-time PCR (qRT-PCR)

Real time RT-PCR analysis was accomplished with CFX manager (Bio-Rad) using the IQ^TM^ SYBR Green SuperMix (Bio-Rad). The primers were constructed (by Primer3plus) based on sequences available in NCBI and the qRT-PCR conditions were optimized (efficiency and sensitivity tests) for each primer prior to the experiment (SI Table S2). Three biological replicates each with triplicate were used for each qRT-PCR analysis. Analysis of negative control reactions (without RT and all reagents except template) confirmed no DNA contamination. The gene expressions were normalized by using *pmp-3* as housekeeping gene.

### Statistical analysis

Significance of differences between treatments was determined using one-way analysis of variance (ANOVA) followed by a post-hoc test (Tukey, *p* < 0.05) in SPSS 12.0KO (SPSS Inc., Chicago, Il, USA). Graphs were prepared in Sigma Plot (Version 12.0).

## Electronic supplementary material

Supplementary Information
