# Supplementary material for: JAK/STAT and TGF-ß activation as potential adverse outcome pathway of TiO2NPs phototoxicity in Caenorhabditis elegans
Source: Sci Rep. 2017 Dec 19;7:17833. doi: 10.1038/s41598-017-17495-8 (PMC5736661; doi:10.1038/s41598-017-17495-8)
Supplement: Supplementary file 1 — Supplementary Information [file 41598_2017_17495_MOESM1_ESM.doc]

**Supplementary Information (SI)**

**JAK/STAT and TGF-ß activation as potential adverse outcome pathway of TiO_2_NPs phototoxicity in *Caenorhabditis elegans***

Hunbeen Kim^1#^, Jaeseong Jeong^1#^, Nivedita Chatterjee^1^, Carlos P. Roca^2a,2b,2c^, Dahye Yoon^3^, Suhkmann Kim^3^, Younghun Kim^4^, Jinhee Choi^1*^

^1^ School of Environmental Engineering, University of Seoul, 163 Seoulsiripdaero, Dongdaemun-gu, Seoul 02504, Korea

^2a^ Department of Bioscience, Aarhus University, 8600 Silkeborg, Denmark

^2b^ Autoimmune Genetics Laboratory, Department of Microbiology and Immunology, KU Leuven - University of Leuven, B-3000 Leuven, Belgium

^2c^ VIB Center for Brain and Disease Research, B-3000, Leuven, Belgium

^3^Department of Chemistry, Center for Proteome Biophysics and Chemistry Institute for Functional Materials, Pusan National University, Busan 46241, Korea

^4^Department of Chemical Engineering, Kwangwoon University, 20 Kwangwoon-ro, Nowon-gu, Seoul 01897, Korea

#: These authors contributed equally.

^*^Corresponding author: Jinhee Choi

Tel: 82-2-6490-2869

Fax: 82-2-6490-2859

E-mail: [jinhchoi@uos.ac.kr](mailto:jinhchoi@uos.ac.kr)


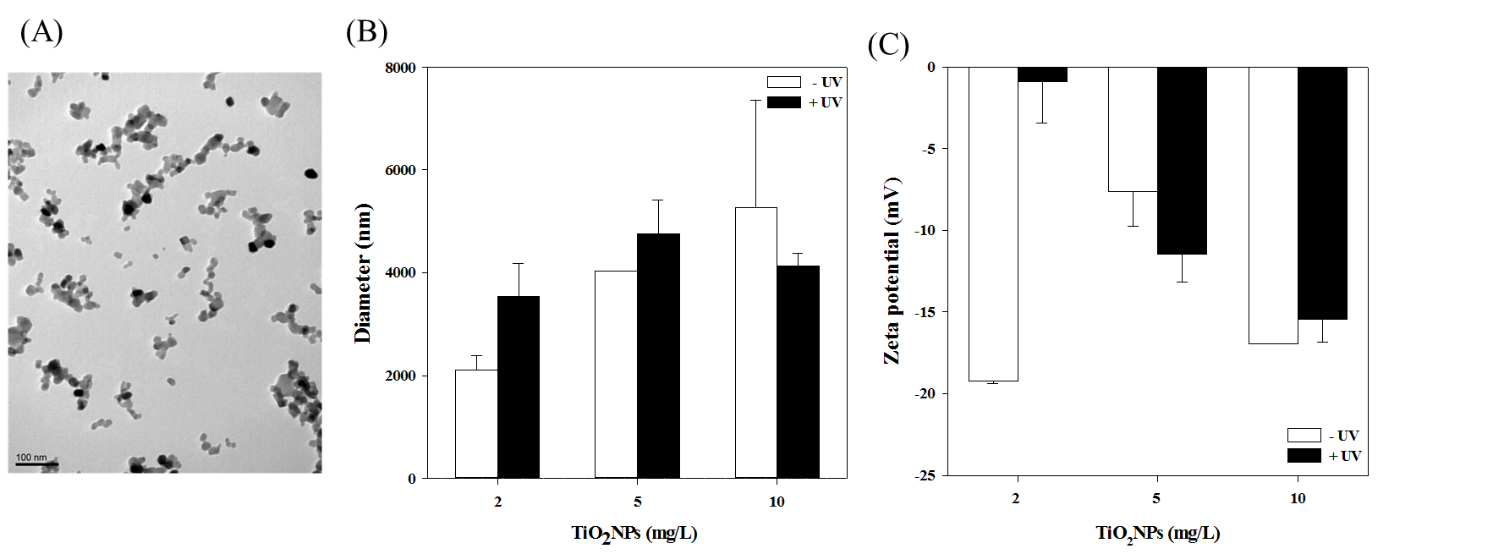


**Fig. S1** Characterization of TiO_2_NPs in K-media. (A) transmission electron microscope (TEM) image of TiO_2_NPs; (B, C) Hydrodynamic diameters of TiO_2_NPs without UV and with UV at 24-h (B), zeta potential of TiO_2_NPs without UV and with UV at 24-h (C).

**Fig. S2.** UV-vis diffuse reflectance spectrum of TiO_2_NPs powder (P25).

**
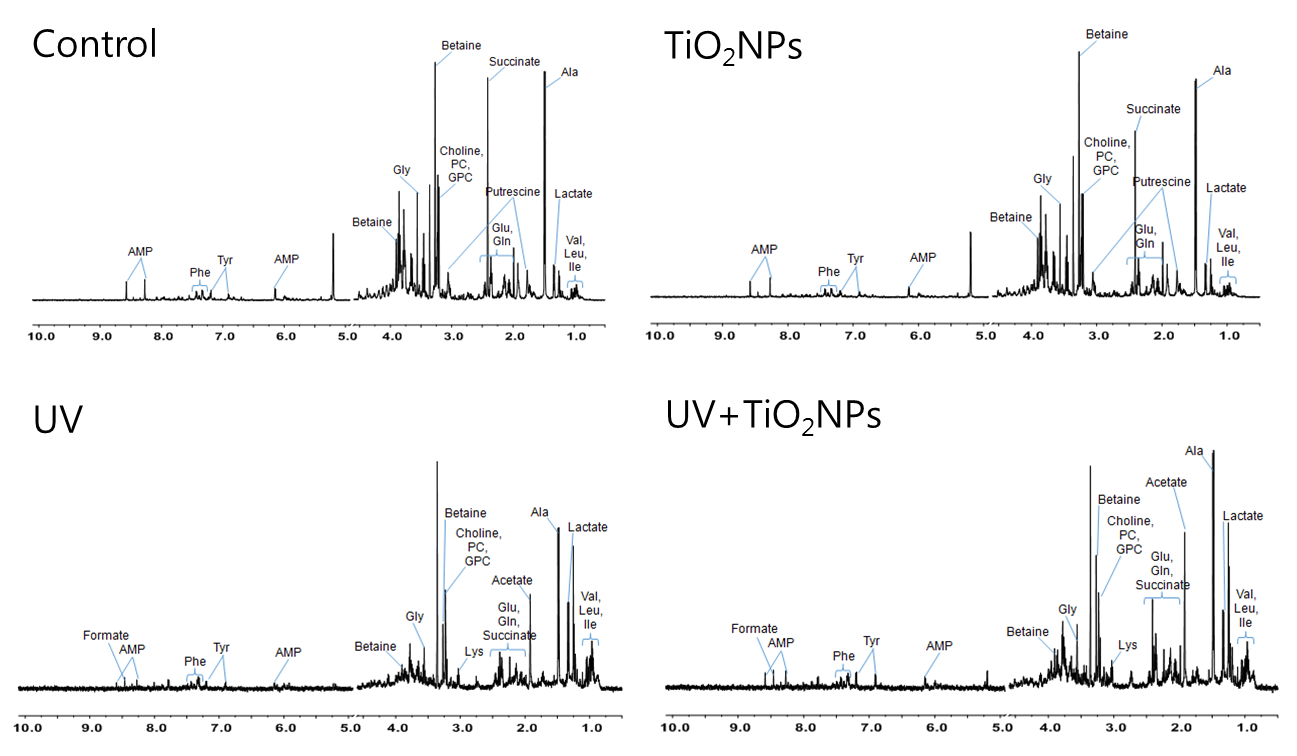
**

**Fig. S3** Global metabolites spectra measured in Control and TiO_2_NPs, UV and UV+TiO_2_NPs exposed *C. elegans* using ^1^H-NMR.

**
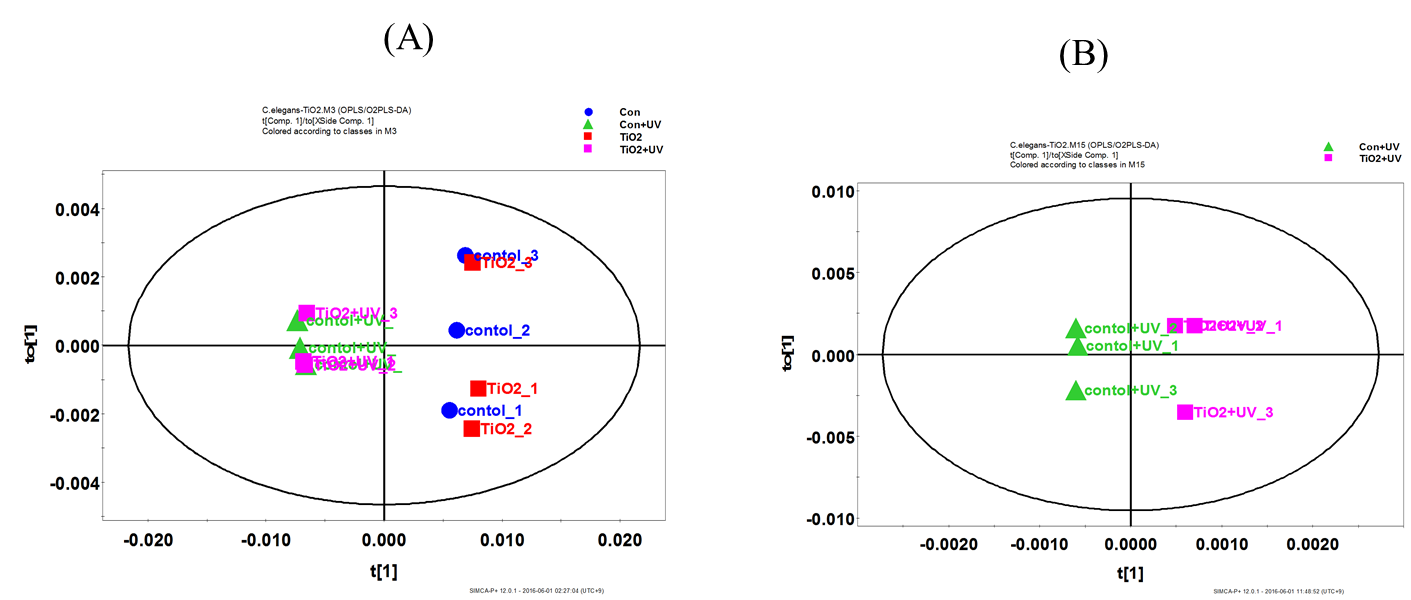
**

**Fig. S4** OPLS-DA score plot from the NMR spectra of metabolomes compared on Control, TiO_2_NPs, UV and UV+TiO_2_NPs (A), and compared on UV and UV+TiO_2_NPs (B).


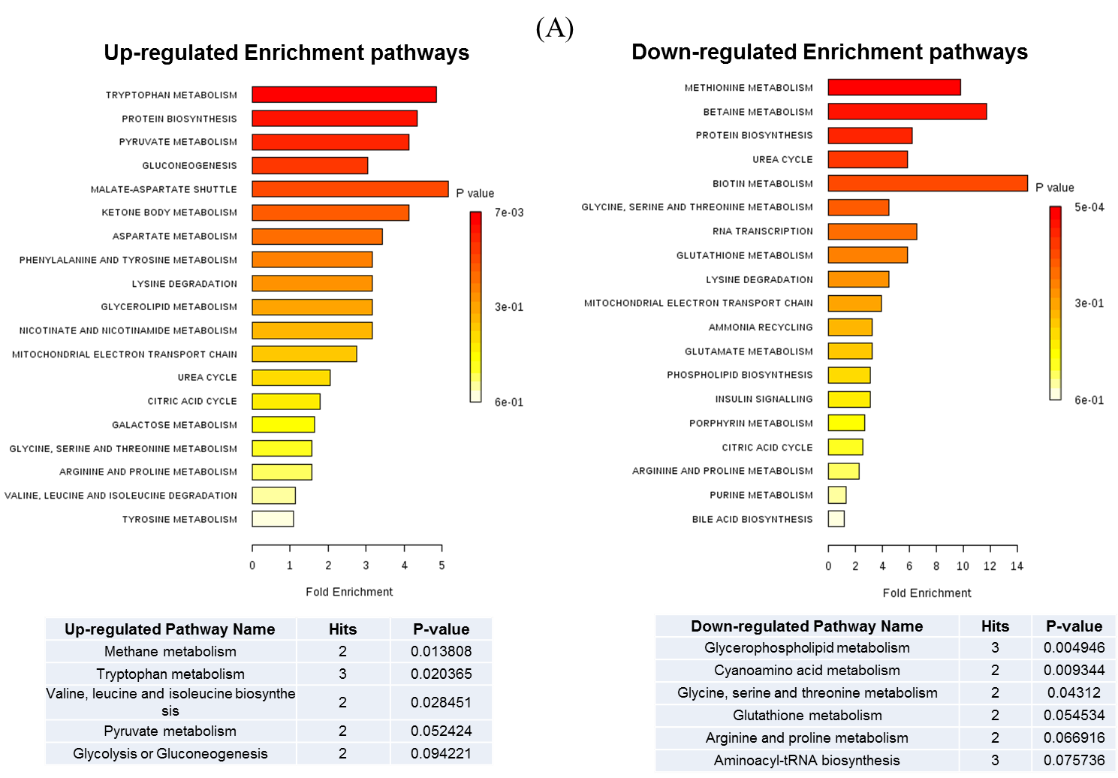


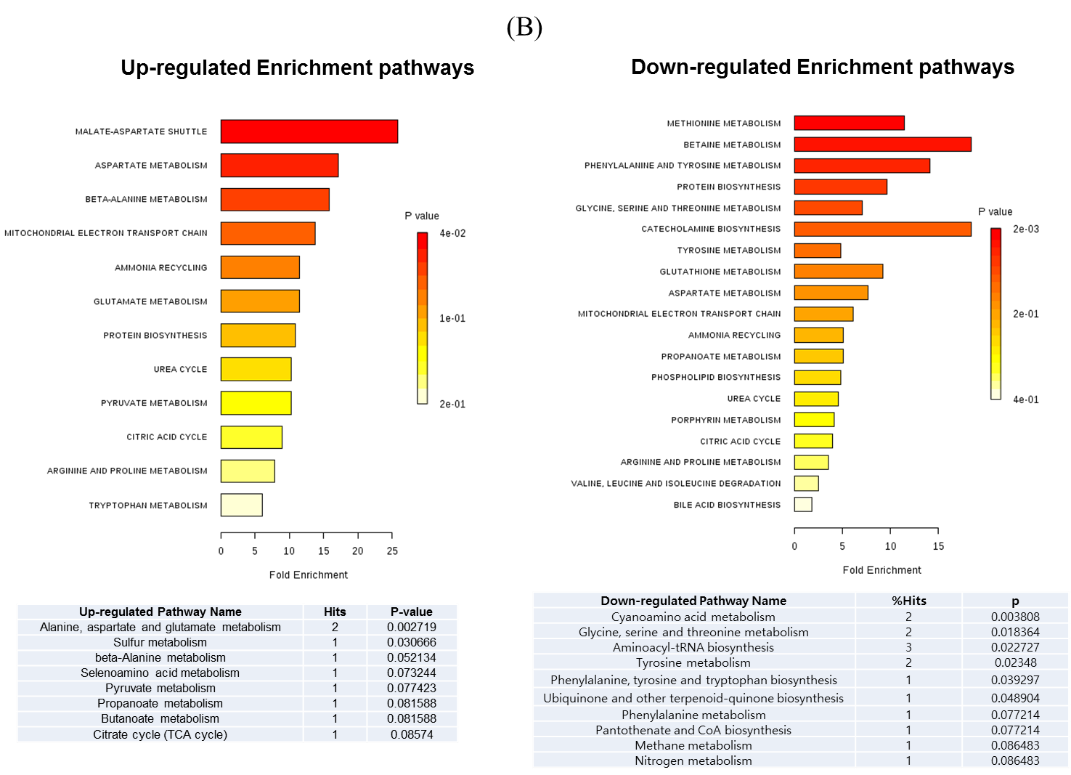


**Fig. S5** Pathway enrichment analysis performed using Metaboanalyst 3.0 based on significantly expressed (*p* < 0.05) NMR metabolites by UV+TiO_2_NPs compared to Control (>1.5 folds, A) and by UV+TiO_2_NPs compared to UV only treatment (>1.5 folds, B).

**Table S1** The mutant strains used in this study. Descriptions are taken from WormBase http://www.wormbase.org/.

| **Genotype** | **Description** |
| --- | --- |
| *daf-1(m40)* | [daf-1](http://www.wormbase.org/search/gene/daf-1) encodes a TGF-beta type I receptor homolog required, in association with the TGF beta-like type II receptor DAF-4, for the regulation of dauer formation by environmental signals through the ASI chemosensory neuron; DAF-1 is bound by BRA-1 and has an intracellular serine-threonine kinase domain; mutations in [daf-1](http://www.wormbase.org/search/gene/daf-1) result in constitutive formation of dauer larvae even in abundant food; DAF-1 is broadly expressed, but expression in the RIM/RIC interneurons mediates development, pathogen avoidance behavior, feeding rate, and quiescence. |
| *daf-5(e1386)* | [daf-5](http://www.wormbase.org/search/gene/daf-5) encodes a proline-rich protein, conserved in *C. briggsae* but not observed in non-nematode genomes, that promotes dauer formation in the group II branch of the dauer pathway, may regulate chemosensation via AWC neurons, and may regulate egg laying; [daf-5](http://www.wormbase.org/search/gene/daf-5) mutations suppress the dauer phenotype of group II Daf-c mutants; [daf-5](http://www.wormbase.org/search/gene/daf-5)([e1385](http://www.wormbase.org/search/variation/e1385)) partially suppresses dauer formation by [aex-6](http://www.wormbase.org/search/gene/aex-6)([sa699](http://www.wormbase.org/search/variation/sa699)) mutants at 26.8 degrees C. |
| *daf-7(e1372)* | [daf-7](http://www.wormbase.org/search/gene/daf-7) encodes a member of the transforming growth factor beta superfamily; in *C. elegans*, DAF-7 functions as part of a signaling pathway that interprets environmental conditions to regulate energy-balance pathways that affect dauer larval formation, fat metabolism, egg laying, pathogen avoidance behavior, and feeding behavior; [daf-7](http://www.wormbase.org/search/gene/daf-7) reporter gene fusions are expressed in the ASI, ADE, and OLQ neurons, and [daf-7](http://www.wormbase.org/search/gene/daf-7) expression is induced in both ASI and ASJ chemosensory neurons in response to the pathogenic bacterial strain Pseudomonas aeruginosa PA14. |
| *sos-1(cs41) V.* | [sos-1](http://www.wormbase.org/search/gene/sos-1) encodes an ortholog of Son of sevenless, a guanine nucleotide exchange factor; [sos-1](http://www.wormbase.org/search/gene/sos-1) is required for viability, sex myoblast migration, vulval induction, and oogenesis; [sos-1](http://www.wormbase.org/search/gene/sos-1) acts genetically downstream of [let-23](http://www.wormbase.org/search/gene/let-23) and upstream of [let-60](http://www.wormbase.org/search/gene/let-60) with respect to vulval development; SOS-1 binds bovine calmodulin in vitro in a calcium-dependent manner. |
| *sem-5(n1779) X.* | [sem-5](http://www.wormbase.org/search/gene/sem-5) encodes a Src homology (SH) domain 2 and 3-containing protein, orthologous to human GRB2 ([OMIM:108355](http://omim.org/entry/108355)) and Drosophila Drk; [sem-5](http://www.wormbase.org/search/gene/sem-5) functions in multiple signaling pathways during development including those regulating sex myoblast migration, muscle membrane extension, vulval induction, fluid balance, viability, and formation of the male tail; SEM-5 acts downstream of the LET-23 epidermal growth factor receptor to negatively regulate RAS-, MAP-, and IP-3-, mediated signal transduction; a [sem-5](http://www.wormbase.org/search/gene/sem-5)::yfp promoter fusion is expressed in many cells throughout development, including the hypodermis, intestine, neurons, body wall muscles, and vulval precursor cells. |
| *sta-1(ok587) IV.* | [sta-1](http://www.wormbase.org/search/gene/sta-1) encodes a protein that is a member of the STAT family of transcription factors; from N- to C-terminus, STA-1 contains conserved coiled-coil, DNA-binding, and SH2 domains, but apparently lacks a conserved amino-terminal oligomerization domain found in other STAT family members; [sta-1](http://www.wormbase.org/search/gene/sta-1) activity is required for repressing dauer formation at high temperatures and genetic analyses indicate that STA-1 acts redundantly with some members of the DAF-7/TGF-beta signaling pathway to repress dauer formation, particularly at low temperatures; tyrosine-phosphorylated STA-1 is able to bind a high affinity mammalian STAT binding sequence, and the STA-1 C-terminus can function as a transcriptional activation domain; [sta-1](http://www.wormbase.org/search/gene/sta-1) is widely expressed during most life stages, including the dauer stage, and is found in the pharynx, intestine, body wall muscles, and in neurons; STA-1 localizes to both the cytoplasm and the nucleus, with expression in the latter found particularly in some amphid neurons; in some neurons, STA-1 expression appears to be negatively regulated by DAF-7/TGF-beta signaling. |

**Table S2** Exposure concentration and duration for each endpoint.

| **Endpoints** | **Exposure condition** | | | **No. of worms** | **Results** | **Rational for exposure level and times** |
| --- | --- | --- | --- | --- | --- | --- |
|  | **Concentration (mg/L)** | **UV** | **Time (h)** |  |  |  |
| Mortality | 0, 2, 5, 10 | With / without | 24 | 20 worms, 8 biological replicates | Fig 1A,B | Range finding test |
| Reproduction | 0, 5 |  | 72 | # of offsprings / individual (150 - 200) | Fig 1D | 5mg/L (LC10) : sublethal concentration  72 hr : optimized time required for *C. elegans* reproduction |
| Oxidative stress | 0, 10 |  | 24 | 20 worms, 8 biological replicates | Fig 2 | 5mg/L (LC10) : sublethal concentration  10mg/L (LC50) : concentration used for rescued effect  24 h : optimized time for molecular level effect measurement |
| Microarray | 0, 5 |  | 24 | Pooled | Fig 3 |  |
| Gene expression | 0, 5 |  | 24 |  | Fig 4 |  |
| Mutant reproduction | 0, 5 |  | 72 | # of offsprings / individual (150 - 200) | Fig 5 | 5mg/L (LC10) : sublethal concentration  72 hr : optimized time required for *C. elegans* reproduction |

**Table S3** Gene and primer lists used for qRT-PCR analysis.

| **Gene** **(wormbase accession No.)** | **Primer sequences** |
| --- | --- |
| *daf-1 (F29C4.1)* | Forward  5' TCAAAACGTGCTGGAATGGC 3' |
|  | Reverse  5' AACAGTTGCTGCCGTTCATC 3' |
| *daf-5 (W01G7.1)* | Forward  5' AGCAGCAAGAATGGAAGG 3' |
|  | Reverse  5' CGCATGTTGTCGAACTCT 3' |
| *daf-7 (B0412.2)* | Forward  5' CGACCAGCTGAACATGAA 3' |
|  | Reverse  5' CCTTCTCCAGTAAGTCCCTA 3' |
| *sos-1 (T28F12.3)* | Forward  5' TCATGCAACAGCCGAATGAG 3' |
|  | Reverse  5' TGTTGTGGCGGATGTTGTTG 3' |
| *sem-5 (C14F5.5)* | Forward  5' GATTTCAACCCACAGGAGAGTG 3' |
|  | Reverse  5' ATTCCACGCCTGTTGTTCAG 3' |
| *sta-1 (Y51H4A.17)* | Forward  5' ACTTGCCCAAATCGGAATGC 3' |
|  | Reverse  5' AGCTGCCAGTTTTGATCAGC 3' |

**Table S4** Metabolism-related pathways analyzed on the DEGs from transcriptomics. (* indicates significant difference at the *p* < 0.05 pathway for each comparison.)

| **Pathway ID** | **Pathway description** | ***p*-value** | | |
| --- | --- | --- | --- | --- |
|  |  | **UV+TiO_2_NPs**  **vs Control** | **UV+TiO_2_NPs**  **vs TiO_2_NPs** | **UV+TiO_2_NPs**  **vs UV** |
| cel00430 | Taurine and hypotaurine metabolism | 2.22E-04* | 2.12E-04* | 1.59E-02* |
| cel00650 | Butanoate metabolism | 2.22E-04* | 2.12E-04* | 1.59E-02* |
| cel00072 | Synthesis and degradation of ketone bodies | 2.22E-04* | 2.12E-04* | 2.38E-02* |
| cel00460 | Cyanoamino acid metabolism | 2.22E-04* | 2.12E-04* | 2.77E-02* |
| cel00910 | Nitrogen metabolism | 2.22E-04* | 2.12E-04* | 2.77E-02* |
| cel00040 | Pentose and glucuronate interconversions | 2.22E-04* | 2.12E-04* | 4.08E-02* |
| cel00130 | Ubiquinone and other terpenoid-quinone biosynthesis | 2.22E-04* | 2.12E-04* | 4.46E-02* |
| cel00250 | Alanine, aspartate and glutamate metabolism | 2.22E-04* | 2.12E-04* | 4.46E-02* |
| cel00260 | Glycine, serine and threonine metabolism | 2.22E-04* | 2.12E-04* | 4.46E-02* |
| cel00410 | beta-Alanine metabolism | 2.22E-04* | 2.12E-04* | 4.46E-02* |
| cel00561 | Glycerolipid metabolism | 2.22E-04* | 2.12E-04* | 4.46E-02* |
| cel00770 | Pantothenate and CoA biosynthesis | 2.22E-04* | 2.12E-04* | 4.46E-02* |
| cel00980 | Metabolism of xenobiotics by cytochrome P450 | 2.22E-04* | 2.12E-04* | 4.46E-02* |
| cel00010 | Glycolysis / Gluconeogenesis | 2.22E-04* | 2.12E-04* | 4.76E-02* |
| cel00830 | Retinol metabolism | 2.22E-04* | 2.12E-04* | 4.76E-02* |
| cel00860 | Porphyrin and chlorophyll metabolism | 2.22E-04* | 2.12E-04* | 4.76E-02* |
| cel00982 | Drug metabolism - cytochrome P450 | 2.22E-04* | 2.12E-04* | 4.76E-02* |
| cel00983 | Drug metabolism - other enzymes | 2.22E-04* | 2.12E-04* | 4.76E-02* |
| cel00051 | Fructose and mannose metabolism | 2.22E-04* | 2.12E-04* | 7.13E-02 |
| cel00330 | Arginine and proline metabolism | 2.22E-04* | 2.12E-04* | 7.13E-02 |
| cel00340 | Histidine metabolism | 2.22E-04* | 2.12E-04* | 7.13E-02 |
| cel00350 | Tyrosine metabolism | 2.22E-04* | 2.12E-04* | 7.13E-02 |
| cel00620 | Pyruvate metabolism | 2.22E-04* | 2.12E-04* | 7.13E-02 |
| cel01200 | Carbon metabolism | 2.22E-04* | 2.12E-04* | 7.13E-02 |
| cel01230 | Biosynthesis of amino acids | 2.22E-04* | 2.12E-04* | 7.13E-02 |
| cel00030 | Pentose phosphate pathway | 2.22E-04* | 2.12E-04* | 7.76E-02 |
| cel00053 | Ascorbate and aldarate metabolism | 2.22E-04* | 2.12E-04* | 7.76E-02 |
| cel00380 | Tryptophan metabolism | 2.22E-04* | 2.12E-04* | 7.76E-02 |
| cel00480 | Glutathione metabolism | 2.22E-04* | 2.12E-04* | 7.76E-02 |
| cel00520 | Amino sugar and nucleotide sugar metabolism | 2.22E-04* | 2.12E-04* | 7.76E-02 |
| cel00630 | Glyoxylate and dicarboxylate metabolism | 2.22E-04* | 2.12E-04* | 7.76E-02 |
| cel01212 | Fatty acid metabolism | 2.22E-04* | 2.12E-04* | 7.76E-02 |
| cel00020 | Citrate cycle (TCA cycle) | 2.22E-04* | 2.12E-04* | 9.18E-02 |
| cel00450 | Selenocompound metabolism | 2.22E-04* | 2.12E-04* | 9.18E-02 |
| cel00500 | Starch and sucrose metabolism | 2.22E-04* | 2.12E-04* | 9.18E-02 |
| cel00640 | Propanoate metabolism - Caenorhabditis elegans (nematode) | 2.22E-04* | 2.12E-04* | 9.18E-02 |

**Table S5** Comparative fold change of the metabolites from NMR based global metabolomics. (Fold change ± Standard error of the mean(SEM))

| **Name of the Metabolites** | **UV+TiO_2_NPs vs Control** | **UV+TiO_2_NPs vs UV** | **UV+TiO_2_NPs vs TiO_2_NPs** |
| --- | --- | --- | --- |
| 2-Aminoadipate | 2.166±0.185 | 1.067±0.074 | 2.677±0.29 |
| 2-Hydroxyglutarate | 1.657±0.076 | 0.923±0.096 | 1.634±0.167 |
| 3-Indoxylsulfate | 2.102±0.456 | 0.903±0.08 | 2.55±1.058 |
| 5-Hydroxytryptophan | 2.818±0.314 | 0.829±0.166 | 2.758±0.892 |
| AMP | 0.505±0.069 | 1.244±0.17 | 0.552±0.069 |
| Acetamide | 0.137±0.005 | 0.541±0.045 | 0.14±0.012 |
| Acetate | 18.478±0.825 | 1.615±0.024 | 18.309±3.172 |
| Acetone | 5.649±0.901 | 1.168±0.161 | 6.764±1.758 |
| Alanine | 0.145±0.002 | 0.346±0.09 | 0.155±0.024 |
| Anthranilate | 2.053±0.02 | 0.73±0.058 | 1.701±0.182 |
| Arginine | 0.515±0.065 | 1.151±0.042 | 0.519±0.026 |
| Asparagine | 1.298±0.312 | 1.312±0.148 | 1.592±0.155 |
| Aspartate | 1.376±0.159 | 2.184±0.355 | 1.649±0.333 |
| Betaine | 0.075±0.002 | 0.561±0.087 | 0.079±0.007 |
| Choline | 0.214±0.063 | 0.545±0.008 | 0.158±0.021 |
| Cystathionine | 1.098±0.138 | 0.853±0.047 | 1.504±0.034 |
| Formate | 4.224±0.27 | 1.344±0.448 | 2.702±0.15 |
| Fumarate | 3.905±0.568 | 0.552±0.177 | 3.576±0.573 |
| Glucose | 1.062±0.009 | 0.724±0.118 | 1.047±0.12 |
| Glutamate | 0.249±0.078 | 1.289±0.043 | 0.242±0.092 |
| Glutamine | 0.719±0.131 | 1.041±0.071 | 0.806±0.184 |
| Glycerol | 2.618±0.679 | 1.432±0.169 | 2.554±0.312 |
| Glycine | 0.151±0.038 | 0.561±0.109 | 0.149±0.001 |
| Indole-3-acetate | 2.559±0.902 | 1.551±0.023 | 2.018±0.273 |
| Isoleucine | 2.228±0.134 | 0.615±0.059 | 2.196±0.17 |
| Kynurenine | 1.811±0.35 | 0.939±0.111 | 1.86±0.398 |
| Lactate | 1.739±0.031 | 0.744±0.171 | 1.7±0.035 |
| Leucine | 2.192±0.084 | 0.679±0.109 | 2.319±0.135 |
| Lysine | 0.489±0.059 | 0.77±0.174 | 0.485±0.121 |
| Malate | 1.842±0.284 | 0.848±0.033 | 3.313±0.336 |
| Methanol | 2.773±0.66 | 1.071±0.208 | 2.943±0.336 |
| Methionine | 1.932±0.557 | 0.781±0.121 | 1.403±0.028 |
| Nicotinate | 2.796±0.814 | 1.128±0.266 | 3.01±0.768 |
| O-Phosphocholine | 0.135±0.063 | 0.852±0.176 | 0.067±0.02 |
| Phenylalanine | 0.676±0.087 | 0.813±0.126 | 0.707±0.073 |
| Putrescine | 0.215±0.009 | 0.918±0.069 | 0.238±0.021 |
| Pyruvate | 1.073±0.145 | 0.336±0.052 | 1.572±0.596 |
| Serine | 0.957±0.124 | 0.992±0.146 | 0.981±0.117 |
| Succinate | 0.376±0.043 | 3.168±0.394 | 0.354±0.014 |
| Threonine | 1.58±0.147 | 1.031±0.186 | 1.501±0.057 |
| Trimethylamine N-oxide | 0.252±0.028 | 0.747±0.217 | 0.24±0.066 |
| Tryptophan | 2.133±0.774 | 0.789±0.019 | 2.376±0.858 |
| Tyrosine | 1.256±0.272 | 0.597±0.051 | 0.604±0.042 |
| Valine | 1.442±0.261 | 0.522±0.114 | 1.409±0.193 |
| sn-Glycero-3-phosphocholine | 0.511±0.001 | 0.839±0.126 | 0.612±0.106 |
